# A Community-Driven Approach to Generate Urban Policy Recommendations for Obesity Prevention

**DOI:** 10.3390/ijerph15040635

**Published:** 2018-03-30

**Authors:** Julia Díez, Pedro Gullón, María Sandín Vázquez, Belén Álvarez, María del Prado Martín, María Urtasun, Maite Gamarra, Joel Gittelsohn, Manuel Franco

**Affiliations:** 1 Social and Cardiovascular Epidemiology Research Group, School of Medicine, Universidad de Alcala, Alcala de Henares, 28871 Madrid, Spain; julia.diez@uah.es (J.D.); pedro.gullon@edu.uah.es (P.G.); maria.sandin@uah.es (M.S.V.); maria.urtasun@uah.es (M.U.); 2 Public Health Institute of Madrid, Madrid City Council, 28007 Madrid, Spain; alvarezsb@madrid.es; 3 Resident of Villaverde, 28021 Madrid, Spain; mfranco@uah.es; 4 Municipal office in Villaverde, Madrid City Council, 28021 Madrid, Spain; gamarrarmt@madrid.es; 5 Global Obesity Prevention Center (GOPC) at Johns Hopkins University, Baltimore, MD 21205, USA; jgittel1@jhu.edu; 6 Department of Epidemiology, Johns Hopkins Bloomberg School of Public Health, Baltimore, MD 21205, USA

**Keywords:** obesity, healthy food, physical activity, urban environment, built environment, Photovoice, citizen science, policy recommendations, community-based participatory research

## Abstract

There is an increasing research interest in targeting interventions at the neighborhood level to prevent obesity. Healthy urban environments require including residents’ perspectives to help understanding how urban environments relate to residents’ food choices and physical activity levels. We describe an innovative community-driven process aimed to develop environmental recommendations for obesity prevention. We conducted this study in a low-income area in Madrid (Spain), using a collaborative citizen science approach. First, 36 participants of two previous Photovoice projects translated their findings into policy recommendations, using an adapted logical framework approach. Second, the research team grouped these recommendations into strategies for obesity prevention, using the deductive analytical strategy of successive approximation. Third, through a nominal group session including participants, researchers, public health practitioners and local policy-makers, we discussed and prioritized the obesity prevention recommendations. Participants identified 12 policy recommendations related to their food choices and 18 related to their physical activity. The research team grouped these into 11 concrete recommendations for obesity prevention. The ‘top-three’ ranked recommendations were: (1) to adequate and increase the number of public open spaces; (2) to improve the access and cost of existing sports facilities and (3) to reduce the cost of gluten-free and diabetic products.

## 1. Introduction

The obesity epidemic has become a major public health concern worldwide [1,2]. In Spain, the prevalence of obesity has been rising steadily over the last decades, increasing from 8.0% in 1987 to 16.5% in 2012 (in adults aged 16 or older) [3]. The persisting health disparities between higher and lower socioeconomic populations have become another key public health issue [4,5,6]. In Spain, previous studies have shown that people of lower socioeconomic status (SES), and people living in lower-SES areas, have higher risks of chronic diseases [7,8]. Social disparities in obesity are particularly large among Spanish women [8]. These larger socioeconomic inequalities in obesity among women have been also identified in other countries of the southern region of Europe [4].

Due to its complexity, single interventions might be sufficient to reduce the obesity epidemic or the socioeconomic health inequities. Traditionally, preventive strategies have targeted high-risk individuals; however, these high-risk approaches have shown modest results, calling for a paradigm shift that addresses the social determinants of obesity [9,10,11]. In this context, there is increasing evidence on the effect that different urban environment characteristics have on obesity [12,13,14]. Moreover, the spatial distribution of obesity can be partially explained by the disproportionate exposure to obesogenic environments [15,16].

The term of “obesogenic environment”, coined by Swinburn et al. [1], relates to the role that environmental factors play in promoting obesity, by shaping both nutrition (e.g., availability of unhealthy foods) and physical activity (e.g., lack of sidewalks). These obesogenic environments contribute to the rising obesity epidemic, in that they influence obesity-promoting behaviors in individuals or populations [17]. As such, there is an increasing focus on how to use policy approaches and how to design and target interventions at the neighborhood level, for instance, to increase physical activity levels, and thereby, in turn, prevent the increase in obesity and overweight [18,19].

Addressing the complexity of obesity can be a significant challenge for policy-makers and for designing interventions with sustained impact, which require the participation of diverse stakeholders [20]. In Spain, the Strategy for Nutrition, Physical Activity and the Prevention of Obesity (NAOS strategy) was designed in 2005 but continues to underscore the relevance of the contextual determinants creating obesogenic environments [21]. Further, most of these guidelines and actions plans do not take into account residents’ perspectives, and usually focus on individual recommendations, (e.g., counseling or health education), where results might be limited or even ineffective [22]. One of the ways for building community support for environmental change is by engaging residents in the research process, as they can provide an insight into the social and environmental conditions that affect their physical activity and their food choices [23]. By including their voice, as Coburn et al. discussed [24], research can promote ‘knowledge democracy’, a situation where knowledge is not restricted to an academic elite but is freely available to all.

Photovoice methodology draws on the notion that residents are the actual experts on their environment and the ones who should guide the actions needed to foster social and policy change at the community-level [25,26,27]. Photovoice is a community-based participatory research methodology, developed by Wang and Burris, that puts cameras into the participants’ hands to help them documenting, reflecting upon, and communicating issues of concern; while stimulating social change and reaching policy-makers [26,28,29]. Thereby, it creates a partnership between researchers and community members, in which the latter is not involved as research subjects, but as co-researchers who actively engage in the entire research process [26,30,31]. Lately, the use of Photovoice in public health research (e.g., to examine the obesogenic environment) has grown [32]. In previous studies, conducted in Canada, Belon et al. [33,34] explored both the food and the physical activity environment using Photovoice. Drawing on their results, they recommended using this methodology to inform the design of community-level interventions, aiming to build healthier environments. Yet, health promotion recommendations are traditionally limited to expert-driven knowledge.

In this article, we describe how we used a community-driven process to translate research findings into environmental policy recommendations for obesity prevention. This project is the result of a unique partnership between an academic research team, public health professionals, local policy-makers and residents of Villaverde, a low-income District in Madrid (Spain).

## 2. Materials and Methods

### 2.1. Research Project Context

This study started with two Photovoice projects, which we conducted in collaboration with the Public Health Institute of Madrid. These projects took place between 2015–2017 in Villaverde, a low-income District located in the southeastern part of the city of Madrid (Spain). These Photovoice projects aimed to identify both environmental facilitators and barriers to residents’ food choices and physical activity [25,35].

We used a purposive sampling strategy to engage participants and based their recruitment on residence location [36]. As key inclusion criteria, we included: (1) living in the neighborhood for more than one year; (2) speaking Spanish; (3) not having impediments to manage a digital camera, and (4) agreeing to attend five group discussion sessions. We used different recruitment strategies (e.g., distributing information sheets, or conducting brief presentations in different neighborhood associations). The resulting sample consisted of 36 participants, which captured images related to their food and physical urban environments. Participants had a mean age of 51.4 years (range 31–72 years). More than half were female and five were foreign-born. Fifteen participants had a low educational level, seven lived with monthly household incomes lower than 900€ (≈982$), and four with monthly household incomes lower than 600€ (≈655$).

Following Wang’s Photovoice methodology [28], we divided the Photovoice process into several sessions [25,35]. First, in an initial session, we explained the project aims and scope and discussed the group sessions schedule with participants. Then, we gave out digital cameras, and participants took part in a workshop, which was led by a professional photographer, who also informed them about the ethics of taking photographs. Then, we invited participants to photograph all the features related to the food/physical activity environment in their neighborhood. We asked them to bring the five photographs that they believed were the most important to the next sessions. Within small discussion groups, participants reviewed and discussed the content and meaning of these photographs with the other group members. Finally, they codified the data and identified the themes that emerged from these data (the photographs and the group discussions) [37]. In this final session, they also selected the photographs to be used in future communication activities. Once the group discussion sessions were completed, participants decided to become actively involved in translating their research findings into concrete policy recommendations to improve their food/physical activity environment.

### 2.2. Study Design

**Figure 1:**
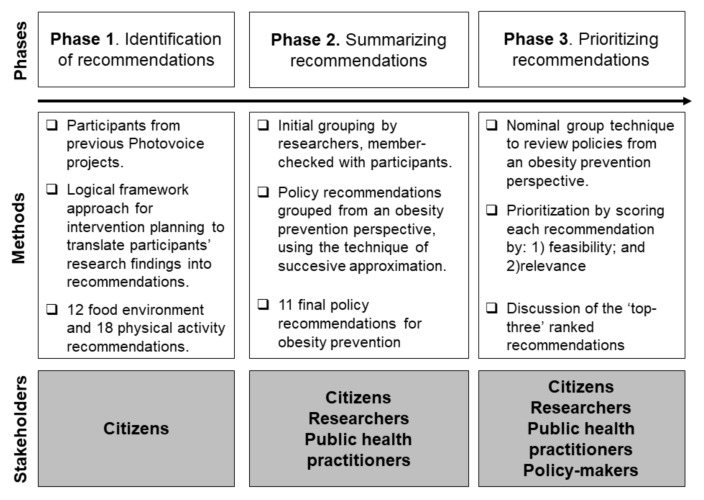
Project overview: Phases of the community-driven process.

We adopted a community-based research approach in this study [38]. Figure 1 provides an overview of the project and of its three different phases. First, based on the findings from the previous Photovoice projects, participants identified a set of policy recommendations. A second phase involved summarizing residents’ recommendations for obesity prevention. Finally, a third phase engaged residents, researchers and community representatives (public health practitioners and local policy-makers) in a nominal group session to review and prioritize these potential recommendations. All individuals completed written consent forms in order to participate in the present study and gave written permission to publish their photographs and data. The study was conducted in accordance with the Declaration of Helsinki, and it was approved by the Ethics Committee of the Universidad de Alcala (CEI/HU/2017/09).

### 2.3. Phase 1: Identification of Policy Recommendations

Phase 1 engaged participants from the previous Photovoice projects. We used an adapted logical framework approach for intervention planning to translate participants’ research findings into policy recommendations [39]. This approach is designed to describe community needs and issues, to identify problems and desired improvements, and to develop solutions to address them.

We used participants’ themes to build an initial problem tree. Like a tree, this initial problem tree consisted of a trunk, roots, tree knots, and branches. While the trunk was the main problem (e.g., *obesogenic food environment*), tree knots were the specific problems (e.g., *insufficient access to variety of food with suitable quality and price*); roots represented the causes (e.g., *low competition between businesses, or shortage of food retailers)*; and the branches represented the effects (e.g., *poor dietary quality*). Once participants identified the negative situations on the problem tree, they reformulated into positive situations. For instance, the issue of ‘*insufficient access to a variety of food with suitable quality and price*’ was converted into a solution, expressed as ‘*promoting small food retailers*’. These positive achievements were objectives. Therefore, Participants then identified ways by which these solutions could be achieved (policy recommendations) (e.g., *reactivating traditional public markets and small retailers*)*.*

### 2.4. Phase 2: Summarizing Recommendations for Obesity Prevention

Upon participants’ agreement on the final set of policy recommendations, we introduced Phase 2. Initially, the academic research team grouped participants’ policy recommendations into broader policy recommendations to prevent obesity, using the deductive analytical strategy of ‘successive approximation’, a method ‘of qualitative data analysis in which the researcher repeatedly moves back and forth between the empirical data and abstract concepts or theories’ [40,41]. We followed a 3-rounds iterative process to ensure participants’ recommendations were accurately represented. In the first round, the academic research team met to summarize participants’ policy recommendations into broader ones. Then they met for a second round, following the same process. In the third round, the academic research team compared recommendations from the first two rounds and agreed about the final set of policy recommendations. This final set of recommendations was then member-checked with participants.

### 2.5. Phase 3: Prioritization Process

Van der Ven et al. created the Nominal Group Technique (NGT), back in 1972, as ‘a structured meeting which seeks to provide an orderly procedure for obtaining qualitative information from target groups who are most closely associated with a problem area (p. 338)’ [42]. As such, we conducted an NGT session in Phase 3 aiming to: (1) allow participants to rank and rate the list of obesity policy recommendations; (2) provide a means to aggregate individual findings, and (3) allow for multiple individual inputs at a single session [42].

Following the NGT methodology, and in order to increase its validity, we purposively recruited different target groups (of different disciplines): (1) academic researchers (experts in epidemiology and public health); (2) public health practitioners (from the Villaverde Health Promotion Centre); (3) local policy-makers (from Madrid City Council); and (4) residents (from the previous Photovoice projects). All previous Photovoice participants (*n* = 36) were invited to participate, with five agreeing to so in Phase 3. An additional 6 individuals were then invited representing the other target groups, taking into account that they were gender-balanced (one male and one female participant from each field of expertise). Finally, a total of 10 individuals participated in this NGT session: two university-based researchers (one male and one female); two public health professionals (one male and one female); one female local policy-maker from the District Council (the male participant could not stay for the NGT session); and five Photovoice participants (three males, and two females).

Initially, we explained all participants the steps of the NGT session, and its objective (what are the most appropriate strategies for preventing obesity at the environmental level in Villaverde*?*), using the findings of the previous Photovoice projects. Facilitators then read the entire list of 11 recommendations and clarified doubts to participants. This ensured that participants were able to understand the meaning of each of the recommendations, thus enabling individuals to make an informed decision when ranking their priorities. After this, we asked participants to select and rank their top preferences from the list. Each person ranked the set of recommendations in order of priority, by scoring each based on (1) relevance and (2) feasibility. Each person had a total of 5 points to allocate the recommendations. We provided participants with a ranking sheet for recording their votes. This stage was completed in silence and the participants did not share or discuss their ideas with one another. Once they ranked the recommendations, the vote was discussed and defended. Thereby, participants had the opportunity to change the individual rating of priorities. Finally, we held a final discussion of the top-three ranked recommendations [42].

We held this NGT session on 12 December 2017, in a room with a U-shaped table in the municipal office of Villaverde. Two of the co-authors (JD and PG) acted as facilitators of this NGT, providing directions to the group, taking notes, and recording participants’ ratings on an Excel spreadsheet. Upon consent, this NGT session was recorded and transcribed. We calculated the sum of the scores for each recommendation and noted whether it was in the top three, allowing for reporting back of the results to participants.

## 3. Results

In this section, we provide the results of the project, following the three different phases. First, we describe the set of potential environmental policy recommendations that emerged from residents’ perspectives. Then the final list of 11 environmental recommendations for obesity prevention is provided. Finally, we refer to the ranked set of policy recommendations.

### 3.1. Policy Recommendations to Promote Healthy Diet and Physical Activity

**Figure 2:**
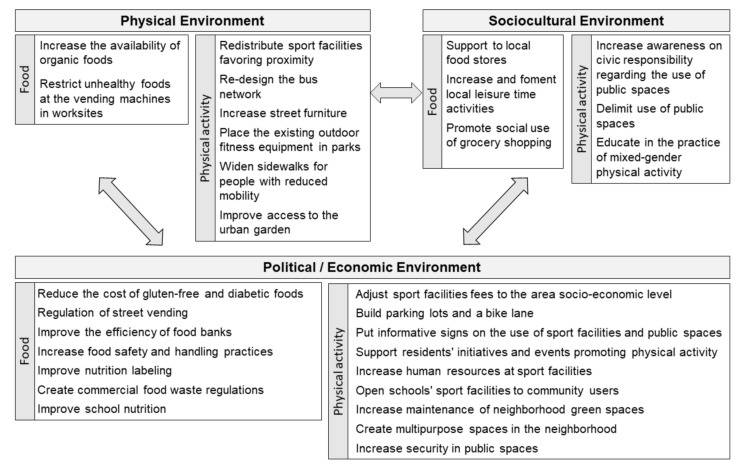
Policy recommendations to support healthy eating and to encourage physical activity.

Through their participatory data analysis, participants (*N* = 36) identified 12 policy recommendations related to their food choices and 18 related to their physical activity. Each recommendation related to one of the categories emerging from the Photovoice process. Figure 2 provides an overview of the entire set of potential physical, sociocultural, economic, and political environmental policy recommendations.

Participants identified 12 recommendations related to their local food environment. Out of the 12 recommendations that emerged from the group discussions, seven were related to the political (e.g., regulation of street vending) and the economic environment (e.g., the cost of gluten-free foods or diabetic products). Concerning the physical environment, participants identified two different recommendations, such as increasing the availability of organic foods in the neighborhood or restricting the wide availability of unhealthy vending machines in their worksites. In relation to the sociocultural environment, they referred to the social meaning of grocery shopping, and to the need to support local small retailers or public markets. They also identified a lack of local leisure facilities in the neighborhood, which they claimed that led residents to spend their leisure time in bars, and thus led them to unhealthy behaviors. Therefore, they stressed the need to offer alternative leisure activities in the neighborhood.

Regarding the recommendations for improving physical activity and mobility, a set of 18 recommendations emerged. Half of these policy recommendations (*N* = 9) were related to the political (e.g., to increase security in public spaces) and to the economic environment (e.g., to adjust sport facilities fees to the area-level socioeconomic status). Regarding the physical environment, they identified six different recommendations (e.g., to redistribute sports facilities, or to increase street furniture as benches in the streets). Finally, residents identified three recommendations related to the socio-cultural environment (e.g., to educate in the practice of mixed-gender physical activity). During group discussions, residents highlighted the importance of using their voice to build the set of recommendations, as well as expressing the feeling of being abandoned by politicians and decision-makers over the last years.

### 3.2. Urban Policy Recommendations for Obesity Prevention

Phase 2 (Figure 1) provided the initial set of recommendations for supporting healthy eating and encouraging physical activity in the neighborhood. Academic research partners met then to collapse similar and overlapping policy recommendations, and to remove those that did not relate to the focus of preventing obesity at the urban environment level (e.g., improving food handling practices). After member-checking them with the Photovoice participants, there were a final total of 11 environmental recommendations for obesity prevention (as shown in Table 1), that were further used in Phase 3.

### 3.3. Ranked Set of Policy Recommendations

**Figure 3:**
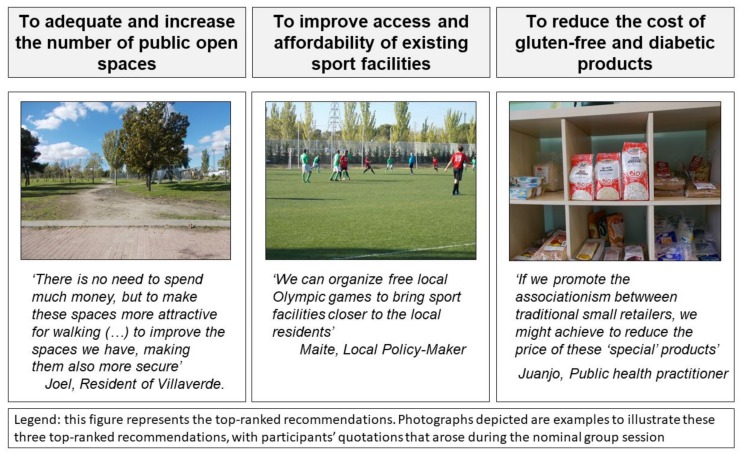
Top-ranked policy recommendations for obesity prevention.

A total of 10 experts took part in a nominal group (NGT) session, including Photovoice participants (*N* = 5), university-based researchers (*N* = 2), public health professionals (*N* = 2), and one local policy-maker from the District Council. The results of this NGT session provided a ranked set of the 11 policy recommendations (shown above in Table 1). The three most rated recommendations (out of the total 11 listed) were, in order of prioritization: (1) to adequate and increase the number of public open spaces offering leisure-time physical activity options; (2) to improve access and affordability of existing sport facilities and (3) to reduce the cost of gluten-free foods and diabetic products. This prioritization process was carried out individually so that experts varied in how they rated the different recommendations. For instance, residents agreed in most of their votes, and they ranked with higher marks the final ‘top three’ recommendations. On the other hand, university-based researchers and public health professionals had higher variability in their ranking. For example, one of the researchers focused on civic responsibility, while the other one highlighted the importance of traditional small neighborhood food-stores. Figure 3 depicts three photographs, which are examples to illustrate each of these three top-ranked recommendations, along with participants’ quotations that arose during the NGT session. The first two photographs were obtained from the physical activity-related Photovoice and the third one from the food environment-related Photovoice. During the NGT session, residents, policy-makers, researchers and public health professionals shared their votes, the agreements and their different point of views of the ranking.

## 4. Discussion

We have described an innovative community-driven process aimed to develop environmental recommendations for obesity prevention in a low-income area in Madrid (Spain). This was a citizen-science project, where residents engaged in collecting, analyzing, and disseminating the research results [43]. This collaborative process allowed participants to identify problems within their community, and to develop these problems into 11 concrete recommendations contributing to prevent obesity.

Residents’ photographs and discussions served as points for departure for an in-depth discussion of the initial 30 policy recommendations (Phase 1 of this study). The value of the Photovoice methodology is not (only) in the photographs itself, but in the group discussions that take place around these photographs. For example, during one of the group discussions, a participant pointed out that he could not afford the cost of some foods for diabetics (e.g., low-sugar food products). This statement initiated a group discussion, where the other participants ended up concluding that the price of some ‘special’ food products (e.g., low-sugar or organic foods) was very high. They claimed that these should be affordable for everyone. This concrete affordability-related recommendation was ranked within the ‘top-three’, which may suggest that successful interventions related to the political/economic environment (such as the affordability of healthy foods) may have a large impact in low-income areas.

Out of this initial set of 30 recommendations that emerged from their group discussions (shown above in Figure 2), others related to the physical (e.g., re-designing the current bus network) or to the sociocultural environment (e.g., supporting local small retailers). Participants suggested interventions that related both to the macro-level (e.g., improving the design of nutrition labels), but also to the micro-level environment (e.g., putting out benches in the streets for the older people), indistinctly. Our results from Phase 1 were consistent with the socioecological ANGELO framework, developed by Swinburn et al. [1], to assess obesogenic environments.

In this study, the community-driven process fostered co-learning between residents and researchers, where both learned from one another [44]. For example, researchers, public health professionals, and the political advisor learned from residents’ insights that the mere presence of a food bank in the neighborhood does not improve healthy food access for those residents in need. Participants’ photographs and discussions illustrated the lack of fresh produce in food banks (e.g., fruits & vegetables). Other issues related to participants’ political/economic environment came up repeatedly. For instance, participants stressed the need to adjust the current sport facilities fees to the area socioeconomic context. This finding illustrates that a sports facility may look great, but that if it is not connected to the residents’ contextual characteristics, it will be difficult for them to use it. Nevertheless, the other top-ranked recommendations were not entirely related to the financial resources of the community, but to the built environment (e.g., the availability of public/green spaces); however, residents perceived that the maintenance of these neighborhood features had been “abandoned” by local authorities in low-income neighborhoods over the last years. In our experience, as presented in the results, residents’ insights allowed to identify key contextual interventions to prevent obesity locally, following Geoffrey Rose’s population approach [9,45]. Their results comprise both scientific and local knowledge.

The methods used during Phase 2 and 3 were successful in translating initial Photovoice results into more concrete recommendations to prevent obesity in the urban environment. Participants took action to make their neighborhood a healthier place. Previous studies examining various stakeholders’ perspectives around obesity, which were conducted in the US [46] and in Mexico [47], showed that the two dominant narratives were related to the ‘personal responsibility’, and to the ‘food environment’. In our study, the central narrative was the ‘physical environment’, very much in line with the domains outlined by Feng et al. [14] as influencing obesity: (1) physical activity facilities (such as parks, sport facilities or playgrounds), and (2) land use and transportation issues (walkability, access to public transport, or mixed land use).

In this context, our results reflect on the importance of physical activity activities to prevent obesity. Residents suggested improving the physical accessibility and affordability of sports facilities, as well as offering new activities to improve physical activity in public open spaces. Both recommendations were ranked as the most important policy recommendations for obesity prevention. Moreover, residents also highlighted the need to improve the streets (e.g., building new bike lanes) as key elements for walking. Implementing car-free streets was another ‘quick’ fix, suggested by participants, which is also amenable to municipal policies. Yet, these recommendations were not ranked within the ‘top-three’. These results illustrate that residents perceived the leisure-time physical activity as being more important for preventing obesity, over transport-related physical activity. We suggest that this may occur because the leisure-time physical activity is associated with higher energy expenditure [48].

The third dimension influencing obesity, as outlined by Feng et al. [14], is the foodscape (in terms of availability of healthy or unhealthy food). Regarding this dimension, we found interesting that residents suggested reactivating traditional retailers (e.g., public markets) and local small businesses (e.g., fishmongers). This concrete recommendation was ranked within the ‘top-five’. Yet, supermarkets were not found to be relevant for residents, contradicting the popular expert-driven suggestion of improving supermarkets’ geographic availability, particularly in low-income communities [46]. In this line, other studies have also linked the increased availability of supermarkets to an increased obesity prevalence [49,50]. This concrete recommendation of reactivating traditional food retailers may be partially explained by the contextual characteristics of Southern-European retail food environments (as the one in Madrid), in which small grocery stores typically offer a large selection of healthy and fresh food (e.g., fruits and vegetables) [51,52,53]. This finding highlights the existing differences across local food environments between cities, or between countries. Therefore, strategies to prevent obesity must take into account the differences in the local obesogenic environment in each area [1,54,55].

As possible implications and directions for further research, we suggest that community-driven processes can bring a series of important benefits for obesity prevention in public health. First, residents’ direct experiences, combined with other stakeholders’ views (such as academics, public health professionals, or local policy-makers), may contribute to adapt existing obesity prevention policies to suit real needs. This draws on the notion that residents are the actual experts on their environment and the ones who should guide the actions needed to foster policy change at the community-level [25,27,56].

Second, this community-driven process connected with a vulnerable community, offering a real opportunity to work with them. Given that obesity affects disproportionally vulnerable populations, we think that interventions that develop community engagement and develop capacity are key to address the health disparities experienced by these vulnerable populations [57]. Moreover, our community-driven process is particularly suitable to build trust and to facilitate residents of vulnerable areas acquiring new knowledge, expanding their social networks, and building new links with different actors (e.g., media) [32,58]. Yet, we acknowledge that the pre-existing tradition of social participation in Villaverde led to the well-functioning group dynamics in this project. We would not expect the same success in group dynamics by doing the same methods in a community without this tradition of social participation.

Third, residents identified key elements of the built environment, that promote healthy eating and active living. Following the Ottawa Charter’s strategies for health promotion, community engagement should be key to create healthier environments [59]. In fact, initiating the process of creating healthier environments only makes senses if it is supported by the community living in the area. It is, therefore, necessary to get the different stakeholders to share their perspectives on building healthier neighborhoods. Yet, policy development is a complex process. In this regard, we provide our approach as an informative process intended to complement traditional policymaking; however, we did not intend to replace traditional expert inputs when developing food policy actions. Further, it should be noted that it is also very important for researchers embarking on these community-driven processes to be clear in advance with participants about the expected outcomes of these projects [60].

According to previous studies, it is critical that people affected by a project stay involved in order to determine its success [61]. In this line, this entire research team study is still committed nowadays to use these results to influence the District policy. As such, we continue working nowadays with local public health practitioners (from the local health promotion center in Villaverde) and meeting with local policy-makers (from Madrid City Council) to implement residents’ policy recommendations in the District. We produced several policy briefs, and we are meeting regularly with local decision-makers (e.g., the bus network authorities) to discuss residents’ recommendations (e.g., modifying the current bus routes in the neighborhood). Additionally, the research team began early to share the project results with the media and the broader community. Participants selected the photographs to be included in the photographic exhibit, but also for the articles that appeared in the newspapers, and for the scientific articles. Further, they have participated in many presentations and dissemination activities of the project results including media interviews, and citizen science meetings at different city-wide forums. Also, residents used these public events as an opportunity to raise awareness on their demands and the scarce resources existing in the District (Villaverde).

### Strengths and Limitations 

There are several limitations to note in our community-driven approach, which are worth mentioning. First, our results, as the results based on any Photovoice project, reflect on the perspective of a given group of participants. Similar to other qualitative methods, the nominal group technique is limited in sample size. Further, we selected participants purposively. As such, both the representativeness and generalizability of our results are limited. However, our process provides an example of a community-driven approach that can be potentially replicated in other communities, neighborhoods, or cities, to develop interventions. We valued transferability over generalizability in this research approach. Second, the policy recommendations were based on residents’ voices; thus, some recommendations might not be based on previous evidence. Third, this collaborative project was a time-consuming process, which may have introduced a selection bias (highly motivated residents would be more likely to participate). Last, we included policy-makers in the last phase of this study; engaging all stakeholders from the beginning could benefit long-term impact and policy follow-up [19]. However, we did not want policy-makers to interfere in the Photovoice sessions, in order to maintain residents’ voices. We argue that participatory methods, like ours, value the knowledge and lived experience of participants, which go beyond what policy-makers see as priorities. By using a citizen science approach to design these obesity prevention policy recommendations, and by using participants’ photographs and discussions to develop the recommendations, our process was uniquely community-driven. Thereby, we have found an effective way to get community input into a policy development process.

Despite these limitations, our results suggest that our community-driven process resulted in the identification of policy issues that were important to residents. Thus, adopting a citizen science approach is key to identify culturally appropriate interventions priorities to prevent obesity [62,63,64]. Adding local knowledge to the existing body of literature may help to respond more effectively to complex societal problems, such as obesity. This approach provided participants with a sense of ownership both over the research and over the outcomes. Further, they were more willing to participate in the dissemination and outreach activities of the findings, which enabled results to reach a wider audience (of both specialized and non-specialized public) [65]. This methodology emphasizes the community perspective over the views of researchers or experts. By means of community-driven approaches, that give participants an active role in knowledge creation and distribution, residents become co-producers of knowledge rather than just being research subjects.

## 5. Conclusions

We described a community-driven approach to translate participants’ research findings into concrete urban environment policy recommendations for obesity prevention. Residents, researchers, public health practitioners and local policy-makers identified 11 concrete policy recommendations for obesity prevention at the urban environment level. Residents ranked as the most relevant recommendations physical activity-related actions, such as improving the physical accessibility and affordability of sports facilities or offering new activities to improve physical activity in public open spaces. They also stressed the need for reducing the cost of some foods (e.g., low-sugar and organic products). This process is the result of a unique and long-standing partnership between an academic research team, public health practitioners, local decision-makers and residents of Villaverde, a low-income district in Madrid (Spain). Community-driven processes might be effective methodologies for identifying sustainable and culturally appropriate health promotion environmental strategies.

## Figures and Tables

**Table 1:** Environmental policy recommendations for obesity prevention.

Action Area	Environmental Recommendation
Physical activity	1. To adequate and increase the number of public open spaces offering leisure-time physical activity options
Physical activity	2. To improve access and affordability of existing sport facilities
Food environment	3. To reduce the cost of gluten-free foods and diabetic products
Physical activity/Food environment	4. Local government involvement in residents’ initiatives promoting physical activity, and in the management of food banks (e.g., facilitating the stock of fresh produce)
Food environment	5. To support small neighborhood food stores and public markets to improve healthy food access
Physical activity/Food environment	6. To increase awareness of civic responsibility regarding the use and maintenance of public spaces; increase awareness on the relevance of school nutrition programs.
Physical activity	7. To improve walkability (e.g., widen sidewalks for people with reduced mobility)
Physical activity	8. To design and build a bike lane
Physical activity	9. To improve public transportation and regulate parking areas
Food environment	10. To improve the design of nutrition labels to promote healthier food options and portion sizes
Food environment	11. To implement healthier options at the vending machines in worksites
